# Determination of the pH Gradient in Hair Follicles of Human Volunteers Using pH-Sensitive Melamine Formaldehyde-Pyranine Nile Blue Microparticles

**DOI:** 10.3390/s20185243

**Published:** 2020-09-14

**Authors:** Dennis Kaden, Lars Dähne, Fanny Knorr, Heike Richter, Jürgen Lademann, Martina C. Meinke, Alexa Patzelt, Maxim E. Darvin, Sora Jung

**Affiliations:** 1 Surflay Nanotec GmbH, 12489 Berlin, Germany; d.kaden@surflay.com (D.K.); L.Daehne@surflay.de (L.D.); 2 Charité–Universitätsmedizin Berlin, Department of Dermatology, Venerology and Allergology, Center of Experimental and Applied Cutaneous Physiology (CCP), 10117 Berlin, Germany; fanny.knorr@gmail.com (F.K.); heike.richter@charite.de (H.R.); juergen.lademann@charite.de (J.L.); martina.meinke@charite.de (M.C.M.); alexa.patzelt@berlin.de (A.P.); sora.jung@charite.de (S.J.)

**Keywords:** triggered drug release, follicular penetration, skin barrier, skin acidity

## Abstract

Nanoparticles can be applied to the hair follicles, which can serve as reservoirs for triggered drug release. A valid measurement method for the determination of the pH within the hair follicle in vivo has not been shown yet. Here, melamine formaldehyde particles up to 9 µm in size were applied on 40 freshly plucked scalp hairs of eight individuals to determine the pH along the hair shaft down to the root area of the hair. For fluorescent pH indicators, pyranine and Nile blue were incorporated into the particles. Measurements were conducted using confocal laser scanning microscopy. A pH decay gradient could be found from the hair sheath towards the external hair shaft (*p* = 0.012) with pH values at the hair sheath of 6.63 ± 0.09, at the hair sheath end at 6.33 ± 0.11, and at the external hair shaft at 6.17 ± 0.09 (mean ± SE). The pH difference between the hair sheath end and the external hair shaft was found to be significant (*p* = 0.036). The results might be comparable with the pH within the hair follicle in vivo indicating a pH increase towards the hair root.

## 1. Introduction

In vivo and ex vivo studies in laboratory animals and humans have demonstrated the roles of hair follicles (HF) as shunt routes for topically applied substances to deeper skin layers [1,2,3,4]. The topical delivery of drugs, such as vaccines, highly potent antibiotics, disinfectants, or DNA particles for transfection to specific follicular target structures, such as the infundibulum (upper part of the hair follicle), the sebaceous gland, the bulge region, and the hair matrix cells (hair root area) [5], may help to treat localized skin disorders selectively with reduced side effects [6]. Moreover, transfollicular delivery of drugs to the capillaries surrounding the hair follicles [7,8] represents a promising option. The properties of nanoparticles can influence their ability to permeate the epidermis and release specifically loaded drugs. Normally, if the skin barrier is intact and the vehicle formulation does not contain any penetration enhancers, nanoparticles are not able to overcome the stratum corneum [9,10]. Particles of 500 nm in diameter or larger, show a specifically efficient penetration into the hair follicles [11]. Once the nanoparticles have entered the hair follicles, these can serve as a reservoir for drug release. The simple application of the nanoparticulate substances as a layer on top of the skin has been shown to be insufficient regarding follicular penetration [12]. Once applied on the skin surface, the particles have to be massaged into the skin to induce a deeper follicular penetration. This effect has been described previously as the ratchet effect. In a corresponding in silico model, the movement of the hair shafts stimulated by massage application and the jagged hair shaft surface have been demonstrated to transport the nanoparticles deep into the hair follicles [13].

Stimuli-responsive drug-loaded nanocarrier (NC) systems that release their drugs in response to specific stimuli are a novel and highly promising prospect. In particular, endogenous triggers for drug release including altered pH or increased temperature gradients in diseased versus healthy skin could help to maximize the concentration of active drugs in the afflicted tissue [14,15]. The acidic pH value of the skin surface (pH 4.2–5.6) and its steep increase across the stratum corneum to 6.8 in the boundary to stratum granulosum and to ≈7.4 in the dermis [16,17] are critical for healthy skin [18,19]. Changes in the pH are reported to play a role in the pathogenesis of skin diseases such as atopic dermatitis, acne vulgaris, and irritant contact dermatitis [20].

Until recently, the physiological pH gradient in the HF was unknown. pH-responsive dendritic polyglycerol nanogels (100 nm–1 µm) conjugated with a pH-sensitive dye and a control dye were recently developed which enabled to determine the pH gradient within the HF of ex vivo porcine ear skin to a depth of ≈530 µm in laser scanning microscopic images of skin sections. The pH gradient in the follicular ducts ranged from pH 6.50 on the skin surface, to pH 7.44 near the point of deepest nanogel penetration, with a sharp increase in pH over the first 300 µm [21]. pH changes corresponding to HF found in diseased skin have not yet been elucidated to date.

In a study conducted by Sahle et al. [22], dexamethasone-loaded pH-sensitive Eudragit^®^ L 100 NC of different sizes was successfully evaluated in vitro using Franz diffusion cells regarding their pH-dependent swelling and erosion kinetics for transfollicular drug delivery. In the present study, a novel method was implemented to determine the pH gradient over the lengths of hair shafts, using plucked hairs from human volunteers and pH-sensitive dyes pyranine (8-hydroxy-1,3,6-pyrenetrisulfonic acid trisodium salt, HPTS) and Nile blue [9-(diethylamino)benzo[a]phenoxazin-5-ylidene]azanium sulfate, NC. The pH values obtained were presumed to correspond to the pH values found in the follicular ducts from which the hairs were removed.

pH-values on defined positions in small volumes are not measurable with typical electrochemical methods due to the lack of space. Micro- or nanoparticles with fitting diameters are able to enter even the smallest of cavities, thus presenting an alternative. In order to use such particles for sensing purposes only an optical readout, preferably in fluorescence, gives sufficiently sensitive data. For the preparation of such sensor particles, several requirements have to be fulfilled:

The particle material has to be easily permeable for H+ ions. This is not given for the usual hydrophobic, polymeric particle materials such as polystyrene or poly(-methylmethacrylate). In preliminary experiments, we have found that the polymer melamine-formaldehyde is able to conduct H+ ions very fast.

A pH-dependent fluorescent dye has to be stably immobilized in the particles without any leaching. Several options, like carboxyfluoresceine, carboxynaphthofluoresceine, and pyranine (hydroxypyrenetrisulfonate) have been tested. Regarding its immobilization, pH-range, and photostability; pyranine was the first choice. In addition, 5-(und -6)-carboxy-SNARF-1 from Thermo Fisher was immobilized showing a pH-dependent ratio between two fluorescence bands. However, in contrast to the free dye in solution, the behavior of the fluorescence bands strongly changed in the polymer, the long emission wavelength band at 640 nm vanished almost completely.

In order to ensure exactly the same fluorescence intensity from particle to particle, they have to be absolutely monodisperse and homogeneously loaded with the fluorescent dye. While the latter could be achieved by careful immobilization during the synthesis, the monodispersity was not sufficiently high. One has to keep in mind, even if the standard deviation in diameter is only 3%, the deviation in fluorescence intensity is already almost 15% due to proportionality to the volume. Hence, a second dye was chosen for ratiometric measurements whose fluorescence intensity has to be pH-independent in the desired pH range and which is not interacting by fluorescence resonance energy transfer with the sensitive dye. These properties are given by the dye Nile blue, which is used in this study.

## 2. Materials and Methods

### 2.1. pH Sensing Melamine Formaldehyde Particles via Fluorescence Measurements of Immobilized Pyranine and Nile Blue

Monodisperse melamine formaldehyde (MF) particles were prepared as described in the literature [23] to 9 µm in particle size. For fluorescent pH sensing, the two dyes pyranine (HPTS) and Nile blue were incorporated into the MF spheres during synthesis. Due to monodispersity, a homogeneous distribution, and the ratio of the dye loading within the particle batch, fluorescence intensity deviations from particle to particle were minimized.

HPTS has been studied as an indicator for optical sensors of pH [24] and is suitable for measurements over the physiological range due to its pKa of 7.3 [25]. Additionally, HPTS exhibits high photostability [26], low toxicity [27], and can be excited at 488 nm, with a fluorescence emission range from 500 to 550 nm.

Nile blue is itself a pH indicator, but due to its pKa of 9.7 [28], there is barely any pH dependence in the physiological range, and additionally, it can be excited beside HPTS at 639 nm without any interaction between the two dyes. Hence, in this case, Nile blue acts as a pH reference dye.

### 2.2. Subjects

At least five hairs were plucked from the scalp of each of the eight healthy volunteers (four male, four female) aged between 32 and 58 years, directly before the measurements using forceps. The volunteers did not have any hair diseases nor stained hairs. On the evening prior to the measurements, the volunteers were asked to wash their hair with a pH-neutral shampoo (Eucerin pH5 Skin Protection Lotion) that was supplied to them.

### 2.3. CLSM Parameters, Calibration, and Hair Measurement

For the measurements, a Zeiss LSM 700 confocal laser scanning microscope (CLSM, Carl Zeiss Microscopy GmbH, Jena, Germany) was used. The following settings were used for all measurements (calibration and subject hairs): pH sensing channel with excitation wavelength 488 nm, emission wavelength 518 nm for HPTS and excitation wavelength 639 nm, emission wavelength 669 nm for the Nile blue reference.

The hair measurements were carried out by putting a recently plucked hair with intact root sheath on a glass slide, then adding 5 µL distilled water as well as 5 µL particle suspension into the drop. A second glass slide was placed on top. CLSM measurements were immediately conducted from the direction of the hair root to the hair tip in different distances between the measurement spots, with several locations on the hair (usually 10 points). All measurements were performed under the same time regime in order to avoid differences by prolonged proton diffusion along the hair during the preparation and measurement.

**Figure 1:**
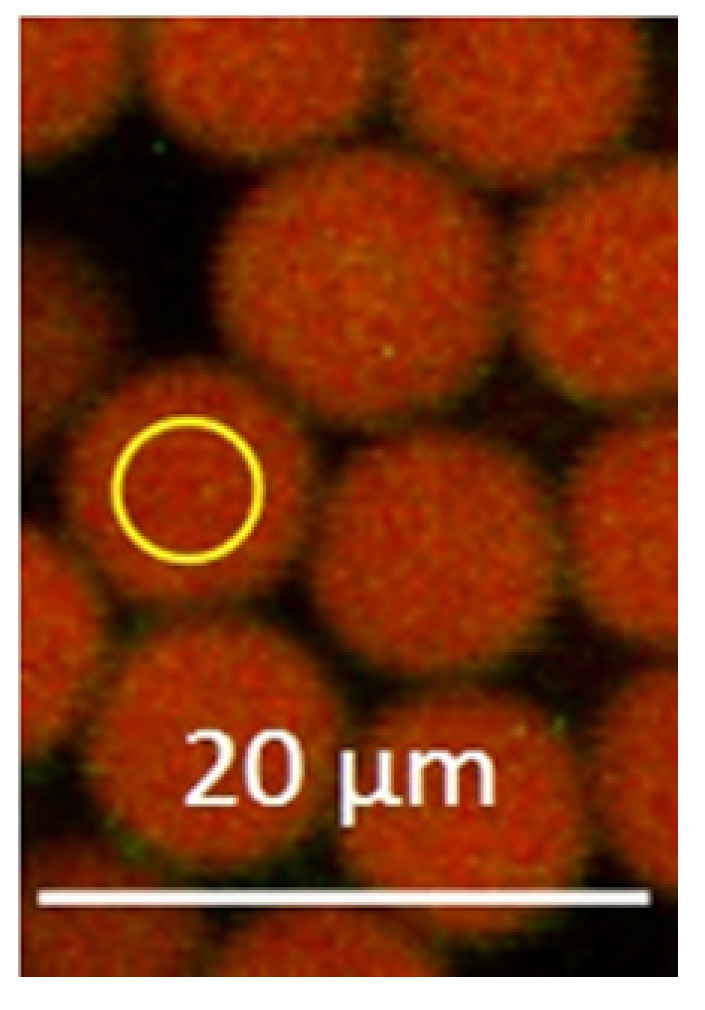
Confocal laser scanning microscope (CLSM) range of interest (ROI) circle inside of fluorescent melamine formaldehyde (MF) particles. Amplification 140×.

The particle measurements were carried out by creating a circular range of interest (ROI) smaller in size than the pictured particles (Figure 1) and moving the ROI inside of randomly chosen particles (three per image). By using a glow over software fluorescence coloring pattern to avoid fluorescence intensity above the detection limit, constant photomultiplier voltages, and the same ROI for each measurement, the results should be comparable. For calculation of the fluorescence intensity, weighted pixel sum values were collected.

The calibration was carried out by applying McIlvaine buffer 0.1 M between pH 4.5 and 7.5 in 0.25 steps (13 calibration points). MF-HPTS-Nile blue beads suspended in water (5% wt.) were provided (5 µL) on a glass slide and were mixed together with 5 µL McIlvaine buffer. For each pH step, two images were taken and for each image, three randomly chosen particles were used for the calculation. The exact pH of the different buffer solutions was measured via Orion Versa Star benchtop meter from Thermo Scientific with a Hamilton Robotics Minitrode pH electrode.

**Figure 2:**
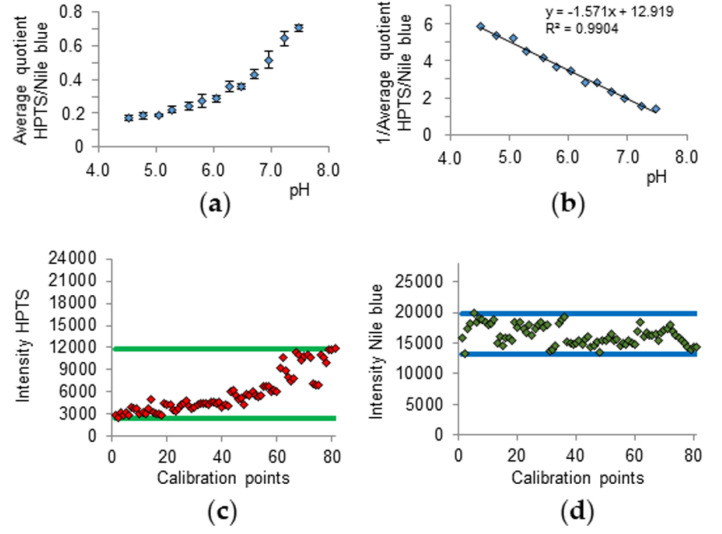
Average quotient of HPTS and Nile blue emission (**a**) its calibration (**b**), HPTS emission intensity raw data points of the calibration (**c**), and Nile blue emission intensity raw data points of the calibration (**d**). Error bars in (**a**) represent the standard deviation of the measurements of three single beads.

In order to evaluate the pH along the hair shaft, the calibration was conducted by measuring particles in McIlvaine buffer between pH 4.5 and 7.5. By forming the quotient of the HPTS and Nile blue emission, the Nile blue referenced pH dependency of HPTS (Figure 2a) can be obtained, whilst forming the reciprocal results in the linear calibration line (Figure 2b).

The pH values of the hair measurements can be calculated from the calibration line (Equation (1)).

(1) $$\mathrm{pH}\;=\left(\;{\left(\frac{\mathrm{Pix}\;\mathrm{Sum}\;\mathrm{HPTS}}{\mathrm{Pix}\;\mathrm{Sum}\;\mathrm{Nile}\;\mathrm{Blue}}\right)}^{-1}-\mathrm{calibration}\;\mathrm{line}\;\mathrm{intercept}\right){\mathrm{calibration}\;\mathrm{line}\;\mathrm{slope}}^{-1}$$

Additionally, the minimum and maximum fluorescence intensity values of both dyes could act as a threshold in between which the subject hair measurement values should be (Figure 2c,d).

### 2.4. Statistical Analysis

Statistical analysis was conducted using SPSS version 22 (IBM Corporation, Armonk, NY, USA). The Friedman-test was used for the acquired pH data with *p* < 0.05 considered to show statistical significance.

## 3. Results and Discussion

**Figure 3:**
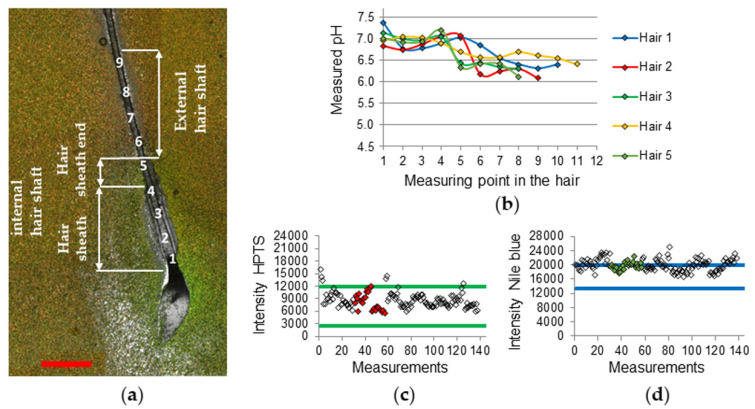
(**a**) CLSM fluorescence overlay of HPTS (green) and Nile blue (red) emission and transmission channels (subject 6, hair 2). Measurement points 1–9. Scale bar: 500 µm. (**b**) pH gradients of the different hairs from subject 6, measurement starts at the hair sheath (points 1–4), then at the infundibulum region (point 5) and ends at the external hair shaft (points 6–9). HPTS emission intensity raw data points of all hair measurements (hair 2 data points are filled) of subject 6 (**c**), Nile blue emission intensity raw data points of all hair measurements (hair 2 data points are filled) of subject 6 (**d**).

The hair measurements from the hair root towards the hair tip included internal hair shaft measurements at several locations at the hair sheath (points 1–4 in Figure 3a), one at the hair sheath end (point 5 in Figure 3a), and several positions along the external hair shaft towards hair tip (points 6–9 in Figure 3a). Points 1–4 include measurements on the bulge and isthmus region, while point 5 on the infundibulum region (Figure 3a).

The pH values for each measuring point were calculated and correlated to the three main regions hair sheath, hair sheath end, and external hair shaft by forming the median value. Figure 3b exemplary shows the pH gradients of subject 6, which all start near pH 7.0 in the area of the hair sheath and end below pH 6.5 at the external hair shaft. This can be clarified visually by Figure 3a, which shows more greenish color in the sheath region, which is evidence for higher HPTS emission intensity that occurs at higher pH values. At the external hair shaft region, the color changes to more reddish, which appears at low emission intensities of HPTS at lower pH values.

The pix sum raw data of subject 6 shows that the HPTS values were mostly inside the calibration threshold, whilst the Nile blue values were located near the upper threshold or above (Figure 3c,d). The reason for this shifting is unclear but could be due to some quenching effect of the calibration buffer, which was absent during the hair measurements. Higher Nile blue values beside normal HPTS values lead to lower pH values and should be considered by interpreting the results.

**Figure 4:**
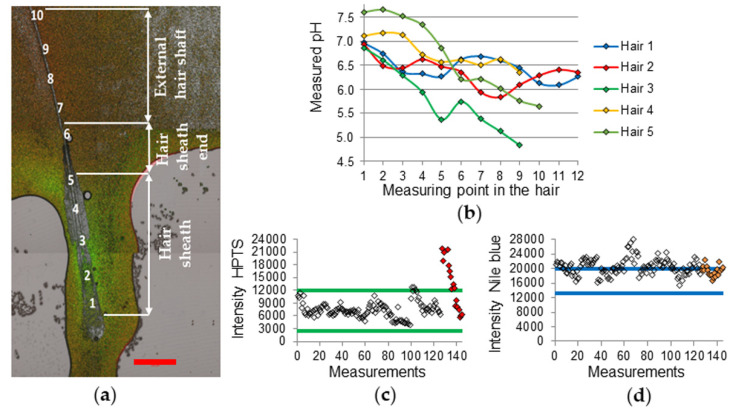
(**a**) CLSM fluorescence overlay of HPTS (green) and Nile blue (red) emission and transmission channels (subject 3, hair 5). Measurement points 1-10. Scale bar: 500 µm. (**b**) pH gradients of the different hairs from subject 3, measurement starts at the hair sheath (points 1–5), then at the infundibulum region (point 6) and ends at the external hair shaft (points 7–10). HPTS emission intensity raw data points of all hair measurements (hair 5 data points are filled) of subject 3 (**c**), Nile blue emission intensity raw data points of all hair measurements (hair 5 data points are filled) of subject 3 (**d**).

In some cases (4 of 40 hairs) even higher pH values than 7.4 and above the calibration limit of pH 7.5 were determined (Figure 4b). These values were always measured in the hair sheath region (Figure 4a). The source of this effect is unknown. The HPTS emission intensity in this region was far above the calibration emission intensities of the dye (Figure 4c). Since every subject should have used the same shampoo, the measuring conditions were comparable in each case. As the effect occurred only in 10% of the measurements, possible HPTS quenching from the calibration buffer which was avoided during the hair shaft measurement seems not to be conclusive. This deviates from the Nile blue emission intensities which are almost all shifted to higher values (Figure 4d), due to possible calibration buffer quenching, which leads to lower pH values.

The obtained pH gradients shown in Figure 3b and Figure 4b are in accordance with pH gradients measured on hair follicles in porcine ear skin biopsy sections using pH-responsive dendritic polyglycerol nanogel [21].

**Figure 5:**
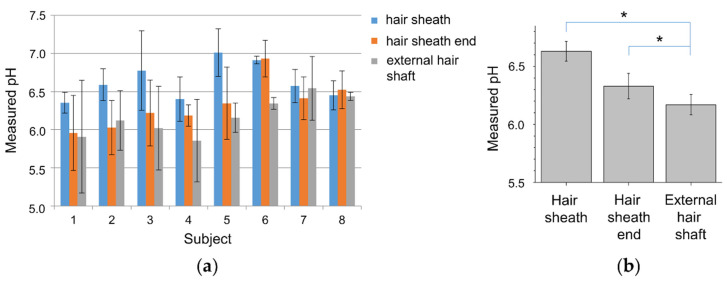
pH values (mean ± SD) of different hair regions summarized via five hair measurements per subject (**a**) and the pH values (mean ± SE) for all subjects in the different hair regions (**b**). “*” represents significant difference *p* < 0.05 between the groups.

By assigning the pH values to the different hair shaft regions, one can distinguish the different pH environments of the hairs. Figure 5 shows this itemized by summarized pH values of hair sheath, hair sheath end, and external hair shaft of five hairs per subject. The subjects 1–6 showed some clear pH shifting from about 6.35 to 7.01 at the sheath region to 5.86 to 6.35 at the external hair shaft. The average difference is about 0.58, is slightly lower than measured for porcine hairs [21]. Beside these results, the subjects 7 and 8 did not show any significant changes, although single hairs showed similar gradient trends.

The mean pH at the hair sheath found was 6.63 ± 0.09, at the hair sheath end at 6.33 ± 0.11, and at the external hair shaft at 6.17 ± 0.09 is lower than reported for porcine hairs [21].

The pH gradients between the hair sheath and the hair sheath end as well as between the hair sheath and the external hair shaft were found to be significantly different (*p* = 0.012). The pH difference between the hair sheath end and the external hair shaft was found to be significant, respectively (*p* = 0.036). The gender-related differences were not found for all hair regions (*p* > 0.05) which is contrary to the published data [29].

Table 1 shows the percentage of hairs of every subject with decreasing pH values from the hair sheath to the external hair shaft (pH difference ≥ −0.5), increasing pH values from the hair sheath to the external hair shaft (pH difference ≥ +0.1), whilst in the remaining hairs, the pH values stagnated. More than 50% of the considered hairs showed a pH decrease ≥ −0.5, whilst approx. 30% of the hairs showed a lower decrease and 12.5% showed an increase.

Possible confounding factors can produce fluctuations in the measured values, which could have led to a wider range of results within the small number of cases of this study. These could be subject-related influences like immense sweating before the measurement changing the salt concentration and eventually affect the measurement otherwise. Confounding factors could also be device-related influences like unstable laser intensity, which would affect the dye fluorescence intensities of indicator and reference as well. This could result in a higher mean variation or even a shifting in the results to higher or lower values, enhanced by using two different laser wavelengths for one measurement with maybe different behavior of the laser stabilities. Time deviations between the duration of the single measurements could lead to inhomogeneous bleaching effects providing different fluorescence emission values from the different dyes. This will be enhanced by using two dyes for one measurement with different bleaching behavior.

This study was designed as a pilot study and for further, future studies a significantly higher number of subjects of different age groups would be desirable in order to deliver optimized results.

By these investigations, the occurrence of a gradient that basically shows a gradient from the hair sheath towards the external hair shaft was, in some cases, clearly shown. On the other hand, there were also cases in which there was hardly a gradient or even increasing pH values towards the hair root. This might be explained by individual variances of each hair, leading to different pH properties.

Furthermore, physiological processes, such as the individual sebum production [30] or the specific phase of the hair cycle, namely the anagen, catagen, or telogen phase [31], of each hair might also influence the pH within the hair follicle.

Other inaccuracies may also influence the pH gradient, such as the hair movement after the application of the particles. The more the particle suspension and the hair move together, the more mixing is to be expected. In addition, the diffusion of the protons over time equalize the pH gradient and weakens the measured gradient. Therefore, it was tried to measure all samples as fast as possible under identical time conditions.

## 4. Conclusions

Using pH-sensitive melamine formaldehyde-pyranine Nile blue microparticles, the present work demonstrates that a pH gradient over the lengths of hair shafts, i.e., follicular ducts, exists. The results at the external hair shaft show pH values close to skin surface pH values (4.2–5.6), while the hair sheath values were shifted in the direction of the blood pH of ≈7.4. Our measurements showed remarkable inter-individual and hair-quality-dependent differences for the pH value of hairs outside the skin. However, in the follicle itself, the deviations were less and the pH value corresponds almost to the physiological pH of 7.2–7.4. Due to this fact, pH-dependent drug delivery and release inside the hair follicles should occur at pH values from 6.5 to 7.0 to ensure the release only inside the follicle.

The developed pH-microsensors are able to accurately measure the pH value in small cavities such as the hair follicle. Moreover, the selected size was well-suited for these special measurements. However, for other applications monodisperse melamine formaldehyde particles in sizes between 200 nm and 12 µm can be synthesized in a monodisperse way with the same kind of dye and loading amount.

## Figures and Tables

**Table 1:** All hair pH measurement results itemized to pH decreasing from hair sheath to external hair shaft (pH difference ≥ −0.5), increasing from hair sheath to external hair shaft (pH difference ≥ +0.1), and pH stagnation (remaining hairs).

Subject	pH Decrease	pH Increase	pH Stagnate
1	40%	20%	40%
2	60%	None	40%
3	60%	None	40%
4	60%	None	40%
5	80%	None	20%
6	100%	None	None
7	20%	60%	20%
8	None	20%	80%
Overall	52.5%	12.5%	35%

## References

[1] Knorr F., Patzelt A., Darvin M.E., Lehr C., Schäfer U., Gruber A.D., Ostrowski A., Lademann J. (2016). Penetration of topically applied nanocarriers into the hair follicles of dog and rat dorsal skin and porcine ear skin. Vet. Dermatol..

[2] Dokka S., Cooper S.R., Kelly S., Hardee G.E., Karras J.G. (2005). Dermal delivery of topically applied oligonucleotides via follicular transport in mouse skin. J. Investig. Dermatol..

[3] Ogiso T., Shiraki T., Okajima K., Tanino T., Iwaki M., Wada T. (2002). Transfollicular drug delivery: Penetration of drugs through human scalp skin and comparison of penetration between scalp and abdominal skins In Vitro. J. Drug Target..

[4] Vandersee S., Erdmenger U., Patzelt A., Beyer M., Meinke M.C., Darvin M.E., Koscielny J., Lademann J. (2015). Significance of the follicular pathway for dermal substance penetration quantified by laser Doppler flowmetry. J. Biophotonics.

[5] Lademann H.J., Richter S., Schanzer M.C., Meinke M.E., Darvin J., Schleusener V., Carrer P., Breuckmann A. (2019). Follicular penetration of nanocarriers is an important penetration pathway for topically applied drugs. Hautarzt.

[6] Hueber F., Wepierre J., Schaefer H. (1992). Role of transepidermal and transfollicular routes in percutaneous absorption of hydrocortisone and testosterone: In Vivo study in the hairless rat. Skin Pharmacol. Physiol..

[7] Blume-Peytavi U., Massoudy L., Patzelt A., Lademann J., Dietz E., Rasulev U., Bartels N.G. (2010). Follicular and percutaneous penetration pathways of topically applied minoxidil foam. Eur. J. Pharm. Biopharm..

[8] Otberg N., Patzelt A., Rasulev U., Hagemeister T., Linscheid M., Sinkgraven R., Sterry W., Lademann J. (2007). The role of hair follicles in the percutaneous absorption of caffeine. Br. J. Clin. Pharmacol..

[9] Baroli B.M. (2010). Penetration of nanoparticles and nanomaterials in the skin: Fiction or reality?. J. Pharm. Sci..

[10] Darvin M.E., König K., Kellner-Hoefer M., Breunig H., Werncke W., Meinke M., Patzelt A., Sterry W., Lademann J. (2012). Safety assessment by multiphoton fluorescence/second harmonic generation/hyper-rayleigh scattering tomography of ZnO nanoparticles used in cosmetic products. Skin Pharmacol. Physiol..

[11] Patzelt A., Richter H., Knorr F., Schäfer U., Lehr C., Dähne L., Sterry W., Lademann J. (2011). Selective follicular targeting by modification of the particle sizes. J. Control. Release.

[12] Patzelt A., Lademann J. (2019). Recent advances in follicular drug delivery of nanoparticles. Expert Opin. Drug Deliv..

[13] Radtke M., Patzelt A., Knorr F., Lademann J., Netz R.R. (2017). Ratchet effect for nanoparticle transport in hair follicles. Eur. J. Pharm. Biopharm..

[14] Döge N., Hönzke S., Schumacher F., Balzus B., Colombo M., Hadam S., Rancan F., Blume-Peytavi U., Schäfer-Korting M., Schindler A. (2016). Ethyl cellulose nanocarriers and nanocrystals differentially deliver dexamethasone into intact, tape-stripped or sodium lauryl sulfate-exposed ex vivo human skin–Assessment by intradermal microdialysis and extraction from the different skin layers. J. Control. Release.

[15] Dong P., Sahle F.F., Lohan S.B., Saeidpour S., Albrecht S., Teutloff C., Bodmeier R., Unbehauen M., Wolff C., Haag R. (2019). pH-sensitive Eudragit^®^ L 100 nanoparticles promote cutaneous penetration and drug release on the skin. J. Control. Release.

[16] Choi E.-H., Man M.-Q., Xu P., Xin S., Liu Z., Crumrine D.A., Jiang Y.J., Fluhr J.W., Feingold K.R., Elias P.M. (2007). Stratum corneum acidification is impaired in moderately aged human and murine skin. J. Investig. Dermatol..

[17] Proksch E. (2018). pH in nature, humans and skin. J. Dermatol..

[18] Fluhr J.W., Elias P.M. (2002). Stratum corneum pH: Formation and function of the ‘acid mantle’. Exog. Dermatol..

[19] Rippke F., Schreiner V., Schwanitz H.-J. (2002). The acidic milieu of the horny layer: New findings on the physiology and pathophysiology of skin pH. Am. J. Clin. Dermatol..

[20] Schmid-Wendtner M.-H., Korting H. (2006). The pH of the skin surface and its impact on the barrier function. Skin Pharmacol. Physiol..

[21] Dimde M., Sahle F.F., Wycisk V., Steinhilber D., Camacho L.C., Licha K., Lademann J., Haag R. (2017). Synthesis and validation of functional nanogels as pH-sensors in the hair follicle. Macromol. Biosci..

[22] Sahle F.F., Balzus B., Gerecke C., Kleuser B., Bodmeier R. (2016). Formulation and in vitro evaluation of polymeric enteric nanoparticles as dermal carriers with pH-dependent targeting potential. Eur. J. Pharm. Sci..

[23] Zhou Y., Yan Y., Du Y., Chen J., Hou X., Meng J. (2013). Preparation and application of melamine-formaldehyde photochromic microcapsules. Sens. Actuators B Chem..

[24] Wolfbeis E.O.S., Furlinger H., Kroneis H. (1983). Fluorimetric Analysis 1. A study on fluorescent indicators for measuring near neutral (physiological) Ph-values. Fresen Z. Anal. Chem..

[25] Offenbacher H., Wolfbeis O.S., Fürlinger E. (1986). Fluorescence optical sensors for continuous determination of near-neutral pH values. Sens. Actuators.

[26] Neurauter G., Klimant I., Wolfbeis O.S. (2000). Fiber-optic microsensor for high resolution pCO2 sensing in marine environment. Anal. Bioanal. Chem..

[27] Lutty G.A. (1978). The acute intravenous toxicity of biological stains, dyes, and other fluorescent substances. Toxicol. Appl. Pharmacol..

[28] Woislawski S. (1953). The spectrophotometric determination of ionization constants of basic dyes1. J. Am. Chem. Soc..

[29] Ping S., Xingquan Z. (1993). Relationship between the elemental content and pH of human hair. Sci. Total. Environ..

[30] Kim M.K., Choi S.Y., Byun H.J., Huh C.H., Park K.C., Patel R.A., Shinn A.H., Youn S.W. (2006). Comparison of sebum secretion, skin type, pH in humans with and without acne. Arch. Dermatol. Res..

[31] Slominski A.T., Paus R., Plonka P.M., Chakraborty A., Maurer M., Pruski D., Lukiewicz S. (1994). Melanogenesis during the anagen-catagen-telogen transformation of the murine hair cycle. J. Investig. Dermatol..

